# Supplementary material for: Unveiling the Bioactive Potential of the Invasive Jellyfish Phyllorhiza punctata Through Integrative Transcriptomic and Proteomic Analyses
Source: Biomolecules. 2025 Aug 4;15(8):1121. doi: 10.3390/biom15081121 (PMC12383608; doi:10.3390/biom15081121)
Supplement: Supplementary file 1 [file biomolecules-15-01121-s001.zip › Suplementary Material/FileS3/PEPFOLD4/XP_065054023_3493/clusters.html]

 PEP-FOLD clusters


Download archive of all models  here   

|  |  |  |
| --- | --- | --- |
| # | file | sOPEP |
| # cluster 1 - Size: 2 |  |  |
|  | XP\_065054023\_3493-model1 | -27.1848 |
|  | XP\_065054023\_3493-model1.1 | -26.8118 |
| # cluster 2 - Size: 2 |  |  |
|  | XP\_065054023\_3493-model2 | -26.8627 |
|  | XP\_065054023\_3493-model2.1 | -20.814 |
| # cluster 3 - Size: 2 |  |  |
|  | XP\_065054023\_3493-model3 | -26.0352 |
|  | XP\_065054023\_3493-model3.1 | -20.7616 |
| # cluster 4 - Size: 1 |  |  |
|  | XP\_065054023\_3493-model4 | -25.9131 |
| # cluster 5 - Size: 3 |  |  |
|  | XP\_065054023\_3493-model5 | -25.264 |
|  | XP\_065054023\_3493-model5.1 | -23.1365 |
|  | XP\_065054023\_3493-model5.2 | -17.7033 |
| # cluster 6 - Size: 2 |  |  |
|  | XP\_065054023\_3493-model6 | -24.7213 |
|  | XP\_065054023\_3493-model6.1 | -24.1385 |
| # cluster 7 - Size: 1 |  |  |
|  | XP\_065054023\_3493-model7 | -24 |
| # cluster 8 - Size: 1 |  |  |
|  | XP\_065054023\_3493-model8 | -23.9762 |
| # cluster 9 - Size: 3 |  |  |
|  | XP\_065054023\_3493-model9 | -23.3196 |
|  | XP\_065054023\_3493-model9.1 | -19.1326 |
|  | XP\_065054023\_3493-model9.2 | -18.245 |
| # cluster 10 - Size: 15 |  |  |
|  | XP\_065054023\_3493-model10 | -23.1526 |
|  | XP\_065054023\_3493-model10.1 | -23.0448 |
|  | XP\_065054023\_3493-model10.2 | -22.8077 |
|  | XP\_065054023\_3493-model10.3 | -22.2796 |
|  | XP\_065054023\_3493-model10.4 | -22.2318 |
|  | XP\_065054023\_3493-model10.5 | -22.2241 |
|  | XP\_065054023\_3493-model10.6 | -22.0979 |
|  | XP\_065054023\_3493-model10.7 | -22.0136 |
|  | XP\_065054023\_3493-model10.8 | -21.9256 |
|  | XP\_065054023\_3493-model10.9 | -21.76 |
|  | XP\_065054023\_3493-model10.10 | -21.5158 |
|  | XP\_065054023\_3493-model10.11 | -21.2399 |
|  | XP\_065054023\_3493-model10.12 | -20.592 |
|  | XP\_065054023\_3493-model10.13 | -19.9964 |
|  | XP\_065054023\_3493-model10.14 | -19.2058 |
| # cluster 11 - Size: 14 |  |  |
|  | XP\_065054023\_3493-model11 | -23.0923 |
|  | XP\_065054023\_3493-model11.1 | -22.8765 |
|  | XP\_065054023\_3493-model11.2 | -22.4487 |
|  | XP\_065054023\_3493-model11.3 | -21.9677 |
|  | XP\_065054023\_3493-model11.4 | -21.9056 |
|  | XP\_065054023\_3493-model11.5 | -21.6487 |
|  | XP\_065054023\_3493-model11.6 | -21.5917 |
|  | XP\_065054023\_3493-model11.7 | -21.3617 |
|  | XP\_065054023\_3493-model11.8 | -21.3119 |
|  | XP\_065054023\_3493-model11.9 | -21.1805 |
|  | XP\_065054023\_3493-model11.10 | -21.0218 |
|  | XP\_065054023\_3493-model11.11 | -20.2192 |
|  | XP\_065054023\_3493-model11.12 | -20.0961 |
|  | XP\_065054023\_3493-model11.13 | -19.6886 |
| # cluster 12 - Size: 2 |  |  |
|  | XP\_065054023\_3493-model12 | -22.7588 |
|  | XP\_065054023\_3493-model12.1 | -21.2393 |
| # cluster 13 - Size: 16 |  |  |
|  | XP\_065054023\_3493-model13 | -22.638 |
|  | XP\_065054023\_3493-model13.1 | -22.5122 |
|  | XP\_065054023\_3493-model13.2 | -22.4936 |
|  | XP\_065054023\_3493-model13.3 | -22.2344 |
|  | XP\_065054023\_3493-model13.4 | -22.2251 |
|  | XP\_065054023\_3493-model13.5 | -22.0808 |
|  | XP\_065054023\_3493-model13.6 | -22.0786 |
|  | XP\_065054023\_3493-model13.7 | -21.946 |
|  | XP\_065054023\_3493-model13.8 | -21.8906 |
|  | XP\_065054023\_3493-model13.9 | -21.6214 |
|  | XP\_065054023\_3493-model13.10 | -21.6051 |
|  | XP\_065054023\_3493-model13.11 | -21.4412 |
|  | XP\_065054023\_3493-model13.12 | -21.1705 |
|  | XP\_065054023\_3493-model13.13 | -20.7553 |
|  | XP\_065054023\_3493-model13.14 | -19.2874 |
|  | XP\_065054023\_3493-model13.15 | -18.1536 |
| # cluster 14 - Size: 2 |  |  |
|  | XP\_065054023\_3493-model14 | -22.0919 |
|  | XP\_065054023\_3493-model14.1 | -20.5682 |
| # cluster 15 - Size: 1 |  |  |
|  | XP\_065054023\_3493-model15 | -21.8718 |
| # cluster 16 - Size: 9 |  |  |
|  | XP\_065054023\_3493-model16 | -21.7853 |
|  | XP\_065054023\_3493-model16.1 | -21.7033 |
|  | XP\_065054023\_3493-model16.2 | -21.2558 |
|  | XP\_065054023\_3493-model16.3 | -21.1643 |
|  | XP\_065054023\_3493-model16.4 | -20.1926 |
|  | XP\_065054023\_3493-model16.5 | -19.5998 |
|  | XP\_065054023\_3493-model16.6 | -19.397 |
|  | XP\_065054023\_3493-model16.7 | -19.2076 |
|  | XP\_065054023\_3493-model16.8 | -18.3267 |
| # cluster 17 - Size: 1 |  |  |
|  | XP\_065054023\_3493-model17 | -21.2899 |
| # cluster 18 - Size: 2 |  |  |
|  | XP\_065054023\_3493-model18 | -21.2369 |
|  | XP\_065054023\_3493-model18.1 | -19.8891 |
| # cluster 19 - Size: 5 |  |  |
|  | XP\_065054023\_3493-model19 | -20.7846 |
|  | XP\_065054023\_3493-model19.1 | -19.3071 |
|  | XP\_065054023\_3493-model19.2 | -19.2902 |
|  | XP\_065054023\_3493-model19.3 | -19.1166 |
|  | XP\_065054023\_3493-model19.4 | -19.0489 |
| # cluster 20 - Size: 1 |  |  |
|  | XP\_065054023\_3493-model20 | -20.5118 |
| # cluster 21 - Size: 2 |  |  |
|  | XP\_065054023\_3493-model21 | -20.4138 |
|  | XP\_065054023\_3493-model21.1 | -18.7331 |
| # cluster 22 - Size: 1 |  |  |
|  | XP\_065054023\_3493-model22 | -20.3431 |
| # cluster 23 - Size: 1 |  |  |
|  | XP\_065054023\_3493-model23 | -20.2801 |
| # cluster 24 - Size: 1 |  |  |
|  | XP\_065054023\_3493-model24 | -19.9701 |
| # cluster 25 - Size: 1 |  |  |
|  | XP\_065054023\_3493-model25 | -19.943 |
| # cluster 26 - Size: 2 |  |  |
|  | XP\_065054023\_3493-model26 | -19.8011 |
|  | XP\_065054023\_3493-model26.1 | -18.2782 |
| # cluster 27 - Size: 2 |  |  |
|  | XP\_065054023\_3493-model27 | -19.5411 |
|  | XP\_065054023\_3493-model27.1 | -19.4425 |
| # cluster 28 - Size: 1 |  |  |
|  | XP\_065054023\_3493-model28 | -19.2794 |
| # cluster 29 - Size: 1 |  |  |
|  | XP\_065054023\_3493-model29 | -19.1072 |
| # cluster 30 - Size: 1 |  |  |
|  | XP\_065054023\_3493-model30 | -18.808 |
| # cluster 31 - Size: 1 |  |  |
|  | XP\_065054023\_3493-model31 | -18.2359 |
| # cluster 32 - Size: 1 |  |  |
|  | XP\_065054023\_3493-model32 | -18.1642 |
|  |  |  |
